# Chemokine Receptor Profiles as Predictors of Survival and Early Progression in Follicular Lymphoma

**DOI:** 10.1002/jha2.70131

**Published:** 2025-08-28

**Authors:** Antonella Zupo, Katrin Pansy, Lukas Gaksch, Julia Waldhart, Andrea Brunner, Johanna Strobl, Marta Szmyra‐Polomka, Sandra Haingartner, Peter V. Tomazic, Hildegard T. Greinix, Barbara Uhl, Julia Feichtinger, Georg Stary, Johannes Haybaeck, Fotini Rosi Vagena, Martin Zacharias, Christine Beham‐Schmid, Peter Neumeister, Katharina Theresa Prochazka, Alexander J. A. Deutsch

**Affiliations:** ^1^ Division of Hematology Medical University of Graz Graz Austria; ^2^ Institute of Pathology Medical University of Innsbruck Innsbruck Austria; ^3^ Department of Dermatology Medical University of Vienna Vienna Austria; ^4^ Department of Otorhinolaryngology Medical University of Graz Graz Austria; ^5^ Division of Cell Biology Histology and Embryology Gottfried Schatz Research Center Medical University of Graz Graz Austria; ^6^ CeMM Research Center For Molecular Medicine of the Austrian Academy of Sciences Vienna Austria; ^7^ Institute of Pathology Medical University of Graz Graz Austria; ^8^ Department of Pathology Saint Vincent Hospital Zams Zams Austria; ^9^ Department of Pathology University Medical Centre Maribor Maribor Slovenia

**Keywords:** chemokine receptors, follicular lymphoma, POD24

## Abstract

**Objective**: Classical follicular lymphoma (FL) is a heterogeneous malignancy. Early progression within 24 months (POD24) is linked to poor outcomes. However, precise risk stratification remains unclear. We aimed to explore chemokine receptor (CR) expression profiles as potential markers of disease biology and outcome in FL.

**Methods**: We analyzed mRNA expression of *CCR1–CCR10*, *CXCR1*–*CXCR5*, *CX3CR1*, and *XCR1* in 52 FL samples (13 POD24, 39 non‐POD24) using RT‐qPCR. Immunohistochemistry for CCR3, CCR7, CXCR3, CXCR4, and CXCR5 was performed. Reactive tonsils (*n* = 5) served as controls.

**Results**: Compared to controls, FL samples showed lower *CCR1*, *CCR6*, *CCR7*, *CXCR1*, *CXCR5*, and *CX3CR1* but higher *CCR4*, *CCR5*, *CCR8*, and *CCR9* expression. Grade 3a FL correlated with reduced *CCR8*, *CXCR1*, and *CXCR3*, and increased *CCR7*. POD24 cases had elevated *CCR3*, *CCR4*, CCR7, *CXCR4*, and *XCR1* but reduced *CXCR3*. High *CCR3*, *CCR4*, and *CCR10* levels were linked to inferior survival. Cluster analysis revealed two CR‐based subgroups; most POD24 cases clustered in the group with worse prognosis.

**Conclusion**: These findings suggest distinct chemokine receptor expression profiles contribute to FL progression. Our data highlight several CRs as candidate prognostic markers and potential therapeutic targets in the context of POD24, warranting further investigation in larger, prospective cohorts.

**Trial Registration**: The authors have confirmed clinical trial registration is not needed for this submission

## Graphical abstract

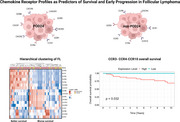

## 1 Introduction

Classical follicular lymphoma (FL) is the most common indolent lymphoma and typically has a favorable prognosis [1, 2]. However, early relapse or disease progression within 24 months of treatment (POD24), which occurs in 20% of chemoimmunotherapy‐treated patients, is linked to poor outcome [3, 4, 5, 6]. Currently, there are no biomarkers to identify POD24‐patients at diagnosis or specific drug targets for this aggressive FL‐subgroup [7].

Chemokine receptors (CRs), a subset of G‐protein‐coupled receptors, mediate processes such as organ development, blood cell formation, immune cell trafficking, and inflammation [8, 9, 10]. B cell homeostatic CRs (*CCR6, CCR7, CXCR3, CXCR4, CXCR5*) are critical for B cell development [11, 12, 13], whereas activation‐induced CRs (*CCR1‐CCR5, CCR8‐CCR10, CXCR1, CXCR2, CX3CR1, XCR1*) play a key role in inflammation [8]. These receptors are also implicated in the development, dissemination, and progression of B‐cell lymphomas [14, 15, 16, 17, 18]. Clinical trials of chemokine receptor antagonists in cancer demonstrate their potential as novel therapeutic approaches [19, 20, 21].

The aim of the study was to exploratively investigate the CR expression profile in our FL cohort and to identify potential associations between CR expression levels, POD24‐status and survival.

## 2 Materials and Methods

### 2.1 Patients and RQ‐PCR

This study included 52 FL‐cases classified according to WHO guidelines [22], comprising 13 POD24‐cases, 21 with relapse after 24 months, and 18 without relapse or progression, and non‐neoplastic tonsil samples, obtained from adults undergoing routine tonsillectomy, serving as controls. All FL‐cases carried the t(14:18) translocation. Clinicopathological details are shown in Table 1. Patients were treated with immunochemotherapy according to the GELF‐criteria [23, 24] at the Medical University of Graz between 2000 and 2015 (last follow‐up: February 2022). Of the 52 patients, 47 were treated immediately, while 5 were treated within two years of diagnosis. A total of 29 patients received an R‐CHOP‐like regimen, and 23 were treated with R‐Bendamustine. FL tissue samples were collected at the time of diagnosis. Progression‐free survival was used to determine POD24‐status, measuring the time from therapy initiation to disease progression, while survival was assessed from diagnosis to lymphoma‐specific death.

**TABLE 1:** Clinicopathologic characteristics of classical follicular lymphoma (FL) patients included in this study. POD24 stands for Progression of Disease within 24 months.

	FL	POD24	non‐POD24
Clinicopathologic parameters	Patients (*n* = 52)	Patients (*n* = 13)	Patients (*n* = 39)
**Sex**			
Male	48% (25)	53.8% (7)	46.2% (18)
Female	52% (27)	46.2% (6)	53.8% (21)
**Age**			
≤60	57.7% (30)	38.5% (5)	64.1% (25)
Male	28.8% (15)	23.1% (3)	30.8% (12)
Female	28.8% (15)	15.4% (2)	33.3% (13)
>60	42.3% (22)	61.5% (8)	35.9% (14)
Male	19.2% (10)	30.8% (4)	15.4% (6)
Female	23.1% (12)	30.8% (4)	20.5% (8)
**FLIPI**			
low	40.4% (21)	23.1% (3)	46.2% (18)
intermediate	25% (13)	23.1% (3)	25.6% (10)
high	34.6% (18)	53.8% (7)	28.2% (11)
**Ann Arbor Stage**			
I	19.2% (10)	7.7% (1)	23.1% (9)
II	5.8% (3)	—	7.7% (3)
III	25% (13)	15.4% (2)	28.2% (11)
IV	50% (26)	76.9% (10)	41% (16)
**Grade**			
1–2	50% (26)	76.9% (10)	53.8% (21)
3a	50% (26)	61.5% (8)	46.2% (18)
**Relapse**			
Yes	65.3% (34)	100% (13)	53.8% (21)
No	32.7% (18)	—	46‐2(18)18
**Survival**			
Dead	32.7% (17)	69.2% (9)	20.5% (8)
Alive	67.3% (35)	30.8% (4)	79.5% (31)

*Note*: n.a. denotes not applicable, because this analysis was just done in lymphoma.

Ethics approval was obtained (28‐040 ex 15/16 and 28–517 ex 15/16), and the study adhered to the Declaration of Helsinki.

RNA extraction, RQ‐PCR, and data analysis were conducted as previously described by our group [15, 16]. Primers for 17 CRs (*CCR1‐CCR10, CXCR1‐CXCR5, CX3CR1, XCR1*) and *GAPDH* serving as a housekeeping gene are listed in Table S1.

### 2.2 Tissue Microarrays and Immunohistochemical Analyses of CXCR3, CXCR5, CCR3, CCR7, CXCR4, and CD68

Tissue microarrays (TMAs) were prepared from 49 FL‐samples and five tonsils. For each case, three histologically confirmed lymphoma tissue cores were analyzed on the TMA. Three cases were excluded due to insufficient material.

Primary antibodies and dilutions for CXCR3, CXCR5, CCR3, CCR7, CXCR4, and CD68 are listed in Table S2 (Supporting Information). IHC was performed as previously described by our group [25]. IHC slides were scanned using the Leica Aperio AT2 system. Staining intensity (0‐3+) and the percentage of positive cells were evaluated over ≥10 high‐power fields (0.242 mm2 each, field diameter: 555.1 µm). Percentages were rounded to 10%. An immunoreactive score (IRS) was obtained by multiplying the percentage of positive cells by the staining intensity divided by 10, according to Zhuang et al. [26].

### 2.3 Multicolor‐Immunofluorescence Staining of T Cells and Subpopulations

Quadruple immunofluorescence staining was performed on FFPE sections using directly labeled monoclonal and secondary antibodies to enhance signal intensity (Table S3). TMAs were stained, counterstained with DAPI, and imaged using an Axio Imager Z1 microscope (Carl Zeiss Inc., Jena, Germany) equipped with an LD Plan‐Neofluar × 20/0.4 objective (Carl Zeiss Inc., Jena, Germany) and TissueFAXS imaging software (TissueGnostics GmbH, Vienna, Austria). Image analysis was conducted using TissueQUEST (TissueGnostics, Vienna, Austria), and quantification was processed in Adobe Photoshop CS5. (Adobe Systems, San Jose, CA).

### 2.4 Statistics, Survival Analysis, Heat Map, and Correlation Plots

Differences in CR expression were analyzed using the Mann‐Whitney U test, with Bonferroni correction for multiple comparisons, and therefore GraphPad Prism (v.10) (GraphPad Software, Boston, MA, USA) was used.

Survival analysis of 17 CR was performed using the R package “survival” [27], with the first quartile as the cut‐off. Kaplan–Meier curves were generated using “survminer” with significance set at *p* ≤ 0.05. The ΔCT values were used to generate the heat map, following an approach similar to that described previously [28, 29, 30]. A heat map with hierarchical clustering (Euclidean distance, Ward linkage) was generated in “ggplot2” [31] applied to scaled data. Correlation plots were created using “CorLevelPlot” [32], with the Spearman correlation.

Cox proportional hazards models were calculated for all the survival analyses performed in this study, using the function “coxph” in the package “survival” [27]. All these analyses were carried out in R (version 4.3.1) [33].

## 3 Results

### 3.1 Substantial Differences in the Expression Pattern of CRs in FLs and Reactive Tonsils

Given the limited knowledge of CRs expression profiles in the progression of FL, we analyzed the expression of 17 CRs (*CCR1–CCR10, CXCR1–CXCR5, CX3CR1*, and *XCR1*) by RQ‐PCR in 52 of FL‐patients (median follow‐up: 8.4 years), all of whom had received immunochemotherapy, and in reactive tonsils (*n* = 5) serving as non‐malignant controls. Patients were grouped into POD24 (*n* = 13), relapsed after 24 months (*n* = 21), and non‐relapsed (*n* = 18). Survival analyses revealed that patients in the POD24‐group had significantly worse cancer‐specific survival compared to the other two groups (hazard ratio of POD24 vs. non POD24: 4.99 [95%CI: 1.66–15.0], *p* = 0.0042; Figure S1a,b,c; *p* < 0.042). No significant difference was observed between the non‐relapsed and relapsed‐after‐24‐months groups (Figure S1d, *p* = 0.054). Notably, lymphoma‐specific deaths occurred in seven out of 13 patients (54%) in the POD24‐group, compared to none in the non‐relapsed group and only six out of 21 patients (29%) in the relapsed‐after‐24‐months group (*p* = 0.0026). Combining the non‐relapsed and relapsed‐after‐24‐months groups into a non‐POD24‐cohort confirmed the worse survival in POD24 (*p* = 0.0015) (Figure S1e, *p* = 0.0015). Notably, POD24‐status was notably not associated with gender, age, FLIPI, grade or Anna stage (Table S4). Thus, the data from our FL‐cohort reflect the major characteristics of other study groups and support the suitability of correlating CR‐expression patterns with POD24‐ and non‐POD24‐status [3, 4, 5, 6].

**FIGURE 1:**
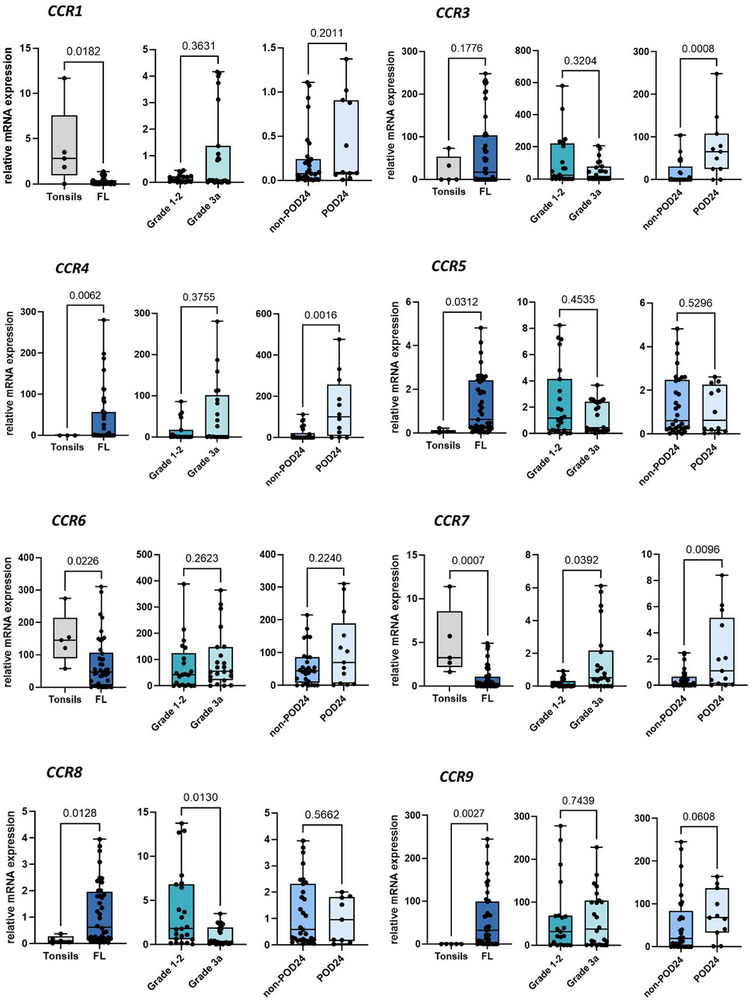
mRNA expression of CCRs in classical follicular lymphoma (FL) and reactive tonsils as healthy control. The box plots show mRNA expression levels of *CCR1*, *CCR3*, *CCR4*, *CCR5*, *CCR6*, *CCR7, CCR8* and *CCR9* in FLs (*n* = 52) and tonsils (*n* = 5), FLs with grades 1–2 (*n* = 26) and with grade 3a (*n* = 26), and non‐POD24 (*n* = 39) and POD24‐FLs (*n* = 13). Values of gene expression were calculated as relative expression. The *p*‐values were calculated by using the non‐parametric Mann–Whitney U test. * denotes B cell homeostatic chemokine receptors (CRs). POD24 stands for Progression of Disease within 24 months.

FLs showed a distinct CR‐expression compared to reactive tonsils, with lower expression of the activation‐induced chemokine receptor *CCR1* (15‐fold decrease, Figure 1; *p* = 0.0182) and the B cell homeostatic CRs *CCR6* and *CCR7* (2.3‐ and 7.1‐fold decrease, Figure 1; *p* < 0.023). In contrast, the expression of activation‐induced CRs *CCR4, CCR5, CCR8*, and *CCR9* were upregulated in FL samples (≥5.9‐fold, Figure 1; *p* < 0.028). Grade 1–2 FLs had 5.8‐fold higher *CCR8* expression than grade 3a (Figure 1; *p* = 0.013). POD24‐FLs expressed ≥2.9‐fold higher levels of the activation‐induced CRs *CCR3* and *CCR4*, as well as the B cell homeostatic receptor *CCR7*, than non‐POD24‐FLs (Figure 1; *p* < 0.01), whereas the activation‐induced CRs *CCR2* and *CCR10* showed no differences (Figure S2).

**FIGURE 2:**
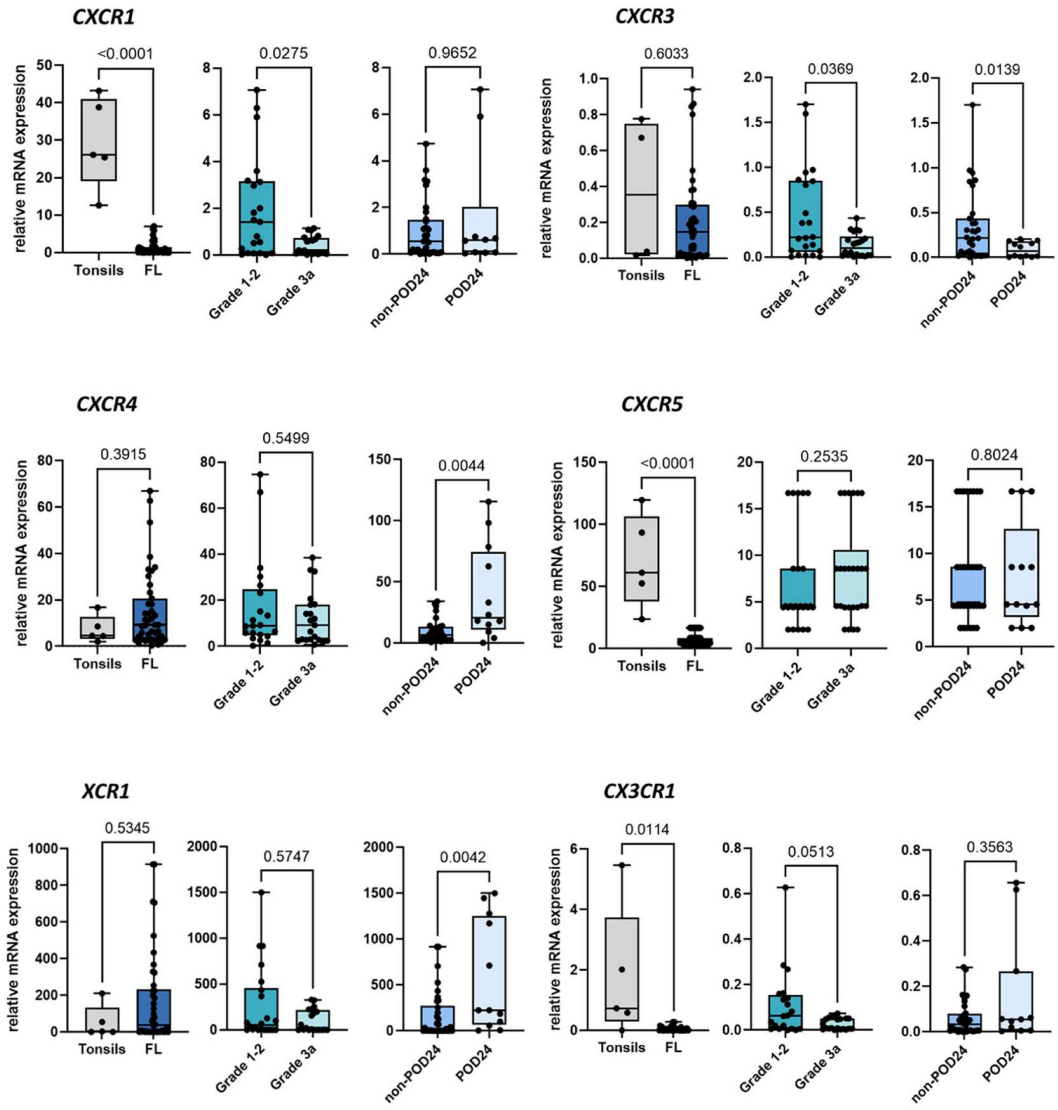
mRNA expression of *CXCRs*, *XCR1*, and *CX3CR1* in classical follicular lymphoma (FL) and reactive tonsils as healthy control. The box plots show mRNA expression levels of *CXCR1*, *CXCR3*, *CXCR4*, *CXCR5*, *XCR1*, and *CCR9* in FLs (*n* = 52) and tonsils (*n* = 5), FLs with grades 1–2 (*n* = 26) and with grade 3a (*n* = 26), and non‐POD24 (*n* = 39) and POD24‐FLs (*n* = 13). Values of gene expression were calculated as relative expression. The *p*‐values were calculated by using the non‐parametric Mann–Whitney U test. * denotes B cell homeostatic chemokine receptors (CRs). POD24 stands for Progression of Disease within 24 months.

In the group of *CXCRs*, the two activation‐induced receptors, *CXCR1* and *CX3CR1*, and the B cell homeostatic receptor *CXCR5* were lower expressed in FLs compared to tonsils (≥13.4‐fold, Figure 2; *p* < 0.001). Interestingly, *CXCR1* and *CXCR3* were ≥ 2.4‐fold higher expressed in grades 1–2 FLs than in grade 3a. POD24‐FLs exhibited 2.4‐fold higher expression of the B cell homeostatic receptor *CXCR4* and the activation‐induced receptor *XCR1* (Figure 2; *p* < 0.04), while the expression of the B cell homeostatic receptor *CXCR3* was 2.5‐fold lower (*p* = 0.0139). No differences were found for the activation‐induced chemokine receptor *CXCR2* (Figure S2).

**FIGURE 3:**
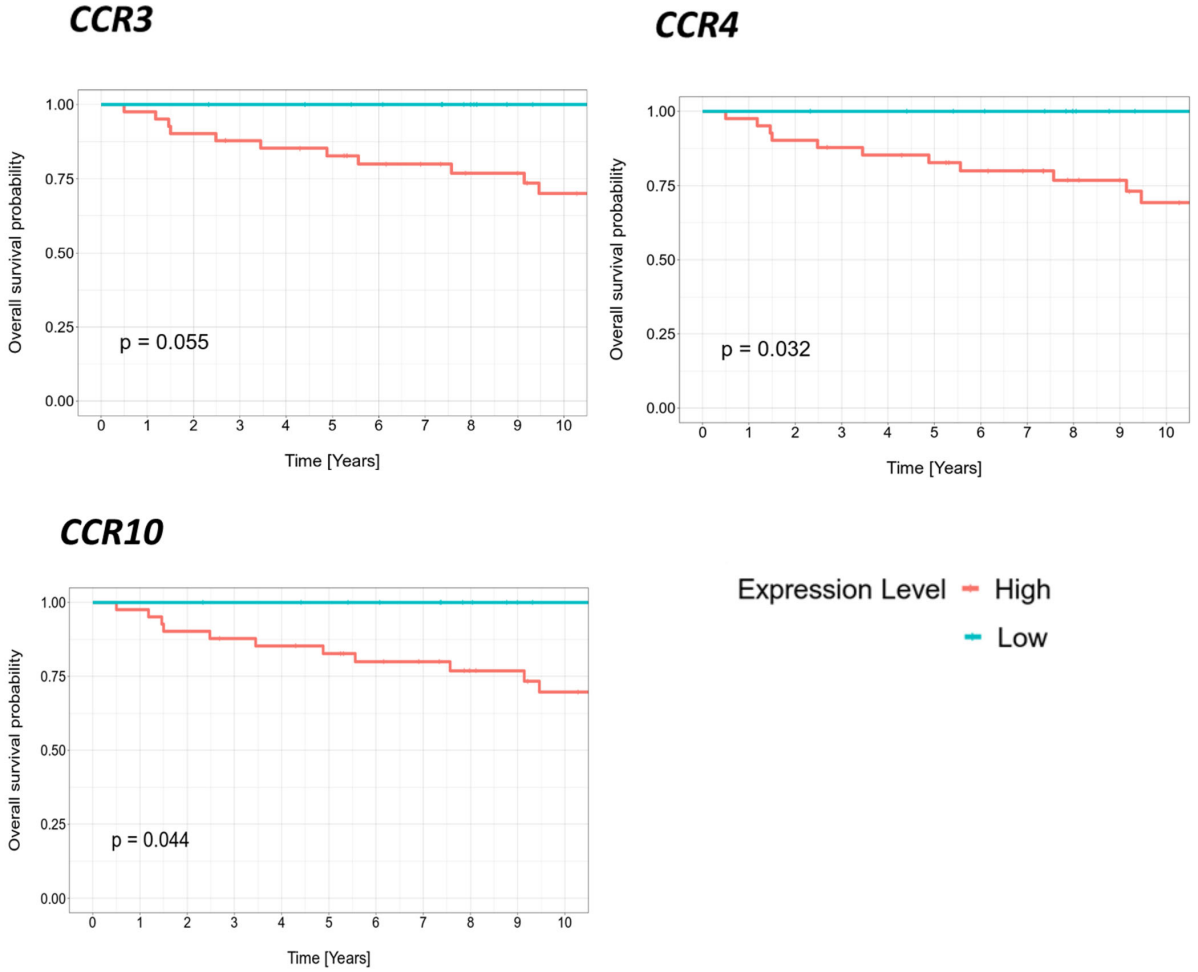
Cancer‐specific survival in relation to chemokine receptor (CR) expression in classical follicular lymphoma (FL) patients. Kaplan–Meier plots show the cancer‐specific survival of our FL patient cohort (*n* = 52; just 10 years are depicted in the plots), which is split into two groups based on the level of *CCR3*, *CCR4*, and *CCR10* expression (high expression in red, low expression in blue, cut‐off is the first quartile).

To assess the relevance of the expression data generated, patients were classified into high‐ and low‐CR expression groups using the first quartile as the cut‐off. High expression of *CCR4* and *CCR10* correlated with poor cancer‐specific survival (Figure 3, *p* < 0.045). In addition, there was a trend toward an association between high *CCR3* mRNA levels and reduced cancer‐specific survival (Figures 3, *p* = 0.055). Notably, no patients in the low‐expression group for these CRs died within the first 10 years.

### 3.2 A Distinct CR Expression Pattern Is Associated With Favorable Cancer‐Specific Survival

**FIGURE 4:**
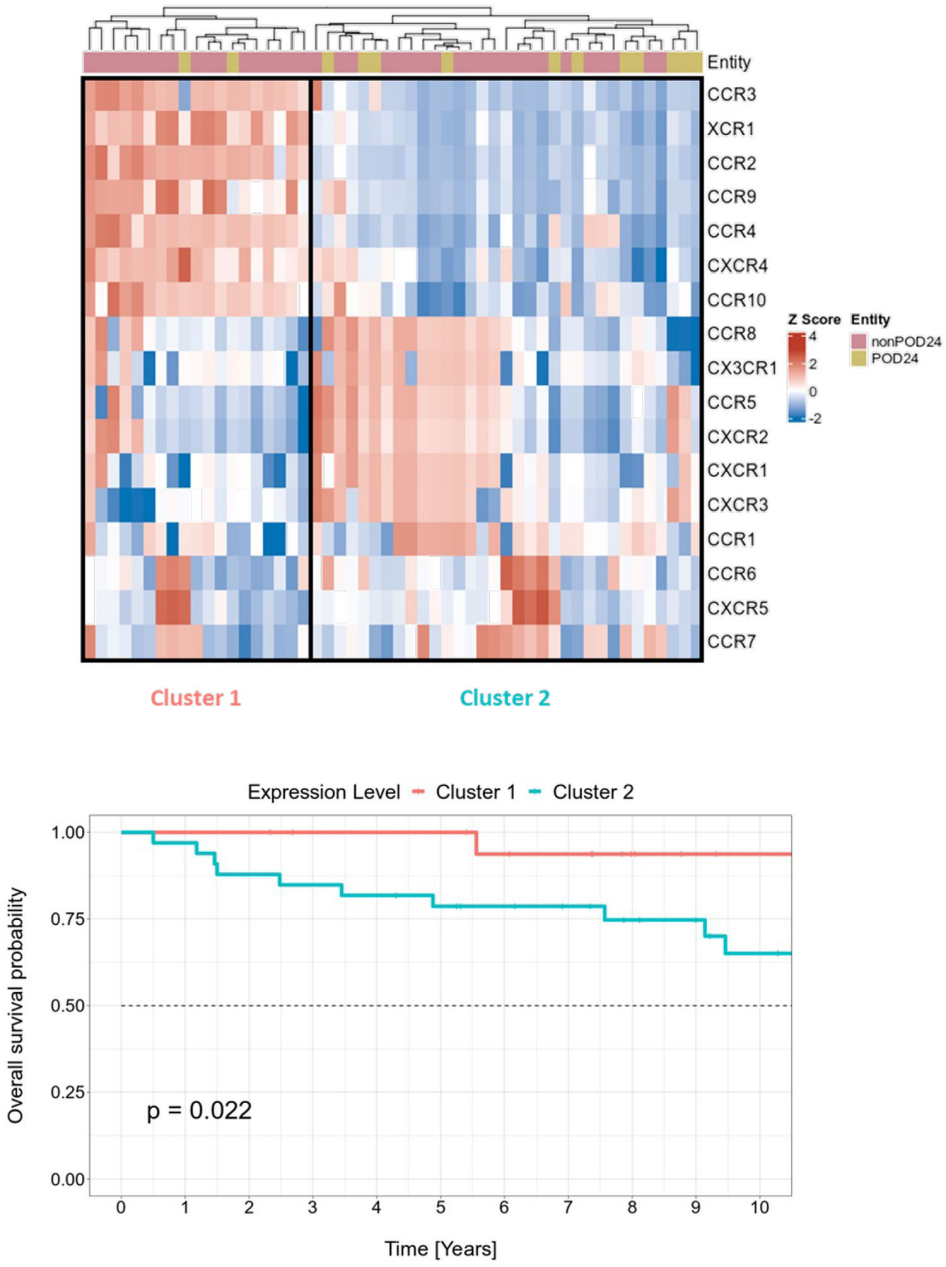
(a) Heatmap based on ΔCT‐values of 17 analyzed chemokine receptors (CR) and hierarchical clustering of classical follicular lymphoma (FL) samples (*n* = 52). The top annotation represents POD24 status: POD 24‐FL cases are depicted in yellow, and non‐POD24 months in pink. Scaled ΔCT‐values have been visualized in the color range of blue and red from lower to higher values. In this case, the lower values (blue) indicate higher mRNA expression and higher values (red) indicate lower mRNA expression. Two discrete clusters are highlighted. (b) Kaplan–Meier plot illustrates the cancer‐specific survival of the patient cohort split into two groups based on cluster analysis (just 10 years of the cancer‐specific survival is plotted). Cluster 1 and cluster 2 are depicted in red and in blue, respectively. POD24 stands for Progression of Disease within 24 months.

To investigate the expression patterns of CR in non‐POD24 and POD24 patients, we performed hierarchical clustering analysis based on mRNA expression using ΔCT‐values. As shown in Figure 4a, the samples were divided into two distinct clusters based on their expression profiles. The first cluster contained almost half of the non‐POD24 samples (17 out of 39) and a small fraction of the POD24 samples (two out of thirteen). In contrast, the second cluster comprised the majority of POD24‐samples (eleven out of thirteen) and more than half of the non‐POD24 samples (22 out of 39), as shown in Table S5. In the first cluster, mRNA levels of half of the activation‐induced CRs (six out of twelve: *CCR2, CCR3, CCR4, CCR9, CCR10*, and *XCR1*) were at least 59.4‐fold lower, whereas three of these (*CCR1, CCR5*, and *CXCR1*) exhibited an at least 3.9‐fold higher expression (Figure S3a,b; *p* < 0.0216). In the group of B cell homeostatic CRs, *CXCR3* mRNA levels were observed to be 5.9‐fold higher in the first cluster compared to the second, whereas *CXCR4* was found to be 10‐fold lower expressed (Figure S3a,b; *p* < 0.0001). Notably, when correlating the cluster analysis with clinical data, we observed a significant association between the first cluster and good cancer‐specific survival (*p* = 0.022, Figure 4b), while no further significant associations were found for the other clinical variables (Table S5). Interestingly, both POD24 patients of the first cluster were still alive at the end of the observation period. These findings suggest that a distinct CR expression pattern may play a critical role in the pathogenesis of FL.

### 3.3 CR Expression Is Not Influenced by Intratumoral Immune Cell Infiltrates

We analyzed intratumoral T cells (CD3+), T helper (CD3+CD4+), and cytotoxic T cells (CD3+CD8+) subpopulations by multicolor‐immunofluorescence staining (*n* = 37), and macrophages (CD68+) by IHC (*n* = 31) in selected cases and correlated the results with mRNA expression (Table 2; Figure S4a). No association was found between immune cell numbers and CR expression, suggesting that lymphoma cells drive the CR pattern (Figure S5a,b). Furthermore, cell content did not differ between non‐POD24 and POD24‐FLs (Table 2).

**TABLE 2:** Immune cell composition and chemokine receptor expression in tumor tissue. This table presents an analysis of intratumoral immune cell populations and chemokine receptor expression in selected cases of follicular lymphoma (FL), focusing on differences based on disease progression within 24 months (POD24). Immune cell subsets, including total T cells (CD3⁺), T helper cells (CD3⁺CD4⁺), cytotoxic T cells (CD3⁺CD8⁺), and macrophages (CD68⁺), were quantified using IHC. In addition, the median percentage and immunoreactive score (IRS) of CCR3, CXCR3, CXCR4, and CXCR5 expression were assessed in reactive tonsils, FL cases without early progression (non‐POD24), and those with POD24.

									CCR3		CCR7		CXCR3		CXCR4		CXCR5	
	CD3+ (Cells/mm^2^)	*p*‐value	CD3+CD4+ (Cells/mm^2^)	*p*‐value	CD3+CD8+ (Cells/mm^2^)	*p*‐value	CD68+ (%)	*p*‐value	%	IRS *‐Median	%	IRS *‐Median	%	IRS *‐Median	%	IRS *‐Median	%	IRS *‐Median
Tonsils (*n* = 5)	n.a.	n.a.	n.a.	n.a.	n.a.	n.a.	n.a.	n.a.	45	6	100	30	27.5	0.25	75	7.5	80	16
Range									(0‐80)		(100)		(0‐80)		(70–80)		(35–80)	
nonPOD24 (*n* = 40)	827.77	0.77	160.26	0.52	267.67	0.74	2.33	>0.999	50	5.5	50	9.75	13.33	4.26	40	4.56	70	12.30
Range	(48.60–5996.22)		(4.71–4744.82)		(0– 3176.56)		(1‐16.66)		(0‐76.7)		(10‐100)		(0‐100)		(20–80)		(20–80)	
POD24 (*n* = 13)	726.56		217.97		288.84		2.33		50	5	50	5	11.84	0.79	40	4	68.33	10.63
Range	(47.65–3794.15)		(3.60–2398.81)		(28.77–973.18)		(1–7.5)		(0‐80)		(10‐90)		(0‐75)		(0–80)		(30–80)	

*Note*: n.a. denotes not applicable, because this analysis was just done in lymphoma.

### 3.4 CCR3, CCR7, CXCR3, CXCR4, and CXCR5 Protein Content Correlates to Their mRNA Levels

**FIGURE 5:**
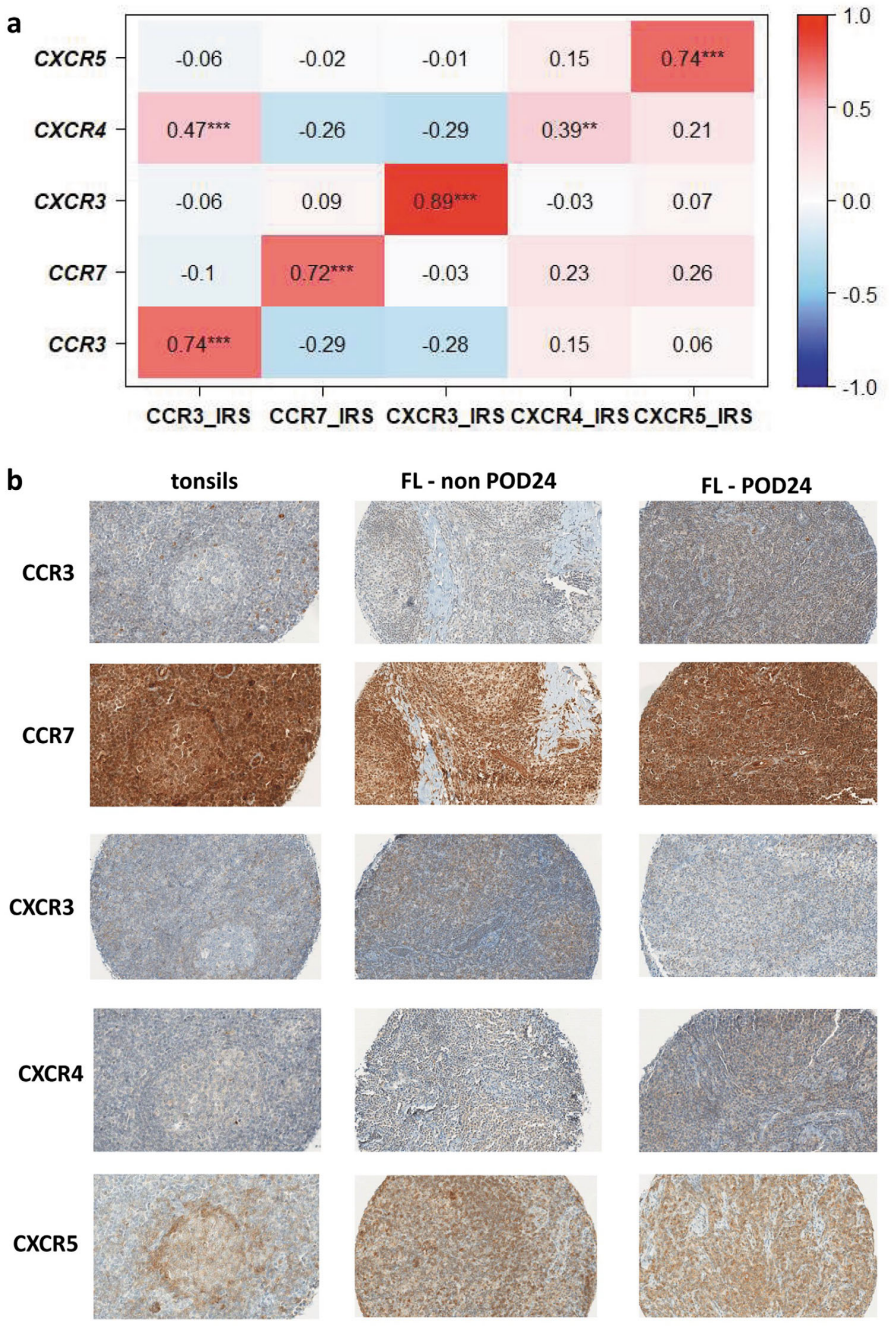
Immunohistochemical analysis performed on CCR3, CCR7, CXCR3, CXCR4, and CXCR5 in classical follicular lymphoma (FL). (a) Comparison of mRNA expression with protein data (IRS); Spearman rho correlation coefficient visualized in shades of blue to red (lower to higher). *p < 0.05, **p < 0.001 and ***p < 0.0001. (b) Representative immunohistochemical stains of CCR3, CCR7, CXCR3, CXCR4, and CXCR5 expression in reactive tonsils, FL non‐POD24 and FL‐POD24. All images were captured using the Leica Aperio AT2 slide scanner (magnification 200 ×). Protein expression levels are reported as IRS values, expressed as the median with interquartile range (Q1–Q3) in brackets. POD24 stands for Progression of Disease within 24 months.

Given the markedly increased mRNA expression of *CCR3, CCR7*, and *CXCR4* and the markedly reduced mRNA expression of *CXCR3* in POD24‐FLs compared to non‐POD24‐FLs, IHC analyses for these four receptors were performed in a selected subset of cases (*n* = 49). In addition, IHC analysis for *CXCR5* was performed as its mRNA levels were significantly lower in FLs compared to reactive tonsils. Protein expression (IRS) correlated with mRNA levels for all receptors (Spearman rho 0.72–0.89; *p* ≤ 0.008, Figure 5a), confirming our RQ‐PCR results.

In reactive tonsils, >75% of germinal center B cells expressed strong CCR7 and CXCR5, with weak CXCR4. Weak CCR3 and CXCR3 staining was seen in 45% and 27.5%, respectively (Figure 5b; Table 2).

In non‐POD24‐FLs, weak CCR3, CXCR3, and CXCR4 expression was observed in 50%, 11.8%, and 40% of lymphoma cells, respectively, while moderate CXCR5 and strong CCR7 expression was detected in 68.3% and 50% (Figure 5b; Table 2).

In POD24‐FLs, weak CXCR3 and CXCR4 expression was found in 13.33% and 40% of lymphoma cells, with moderate CCR3 and CXCR5, and strong CCR7 in 50%, 70%, and 50%, respectively (Figure 5b; Table 2).

## 4 Discussion

This study exploartively investigates the expression patterns of CRs in FL, given their role in lymphomagenesis [14, 15, 16, 17, 18, 25, 34, 35, 36]. CRs play critical roles in the immune system and tumorigenesis by activating pathways that promote tumor cell survival, proliferation, and migration [19, 20, 37]. Data on CR expression in FLs, especially activation‐induced receptors, remain limited.

Our analysis revealed distinct CR‐expression differences between FL‐samples and reactive tonsils, which served as non‐neoplastic controls. FLs showed lower expression of three activation‐induced (*CCR1, CXCR1, CX3CR1*) and three B cell homeostatic CRs (*CCR6, CCR7, CXCR5*) and higher expression of four activation‐induced receptors (*CCR4, CCR5, CCR8, CCR9*) compared to reactive tonsils. These activation‐induced receptors have been reported in various lymphomas [18, 38, 39, 40, 41, 42] and transformed FL [25]. Down‐regulation of *CCR6*, *CCR7*, and *CXCR5* in FLs has also been reported [39, 40]. While CCR1 protein expression was previously found to be upregulated in FLs [38]. This discrepancy may be due to the use of reactive tonsils as controls in our analysis. Overall, our findings support a key role for CRs in the development of FL.

We also found higher expression of *CCR8, CXCR1*, and *CXCR3* in FL grades 1–2 compared to grade 3a. As FL grade 3a harbors more genetic alterations [43], including MYC and TP53 mutations, it may be less dependent on signals from the tumor microenvironment. This reduced dependence could explain the lower CR‐expression, suggesting that aggressive FL subtypes are driven by intrinsic genetic factors.

In our comprehensive analysis, we examined the CR expression pattern in relation to the POD24 status of our FL cohort. Patients with POD24 showed a reduced cancer‐specific survival, confirming findings from other studies [3, 4, 5, 6] and validating the suitability of our cohort for this analysis. POD24 cases showed increased expression of three activation‐induced CRs (*CCR3, CCR4*, and *XCR1*) and two B cell homeostatic receptors (*CCR7* and *CXCR4*), but decreased expression of the B cell homeostatic receptor *CXCR3*. *CCR3, CCR4*, and *XCR1* activate survival pathways upon ligand binding [44, 45, 46], suggesting a role in early progression. *CCR4* may also contribute to the establishment of immunosuppressive conditions in the tumor microenvironment [47]. The B cell homeostatic receptors, CCR7 and CXCR4, contribute to the formation of a supportive niche for lymphoma cells [14, 25, 36, 48], promoting tumor survival, proliferation, and early relapse. Consequently, they emerge as key drivers in the aggressive nature of POD24‐FL. Moreover, *CXCR3* plays a role in mediating the interaction between T cells and lymphoma cells [49, 50]. The reduced expression of CXCR3 in POD24‐FL may alter T cell‐lymphoma interactions, potentially affecting disease progression.

Our survival analysis revealed that high expression of *CCR3*, *CCR4*, and *CCR10* is associated with poor cancer‐specific survival in FL‐patients, a finding that has not been reported previously. These receptors activate pathways that promote cell survival and proliferation [44, 45, 51], suggesting that their elevated expression may lead to more aggressive disease behavior and worse outcomes.

We also observed a low abundance of immune cells (CD3+CD4+, CD3+CD8+, and CD68+ cells) in FLs, which is consistent with previously published findings [52, 53]. Our indicated analysis found no significant impact on FL‐progression, but more detailed investigations are needed to fully understand the role of immune cells in FLs.

Our immunohistochemical analysis revealed variable levels of CCR3, CCR7, CXCR3, CXCR4, and CXCR5 expression in all FL‐subgroups, consistent with previous findings [18]. Protein levels correlated with mRNA expression, and most of these receptors were most highly expressed in POD24 cases, suggesting their potential as biomarkers for risk stratification. Given existing CR‐targeting drugs [19, 20, 21], these may also serve as therapeutic targets for POD24‐FLs.

Our cluster analysis of CR‐expression showed that cluster 2, with higher expression of activation‐induced CRs, had worse clinical outcomes. Notably, the overall CR expression pattern across samples was heterogeneous, which may mirror the underlying biological diversity of FLs. As these CRs respond to inflammatory cytokines, and aggressive FL is linked to elevated inflammatory markers [54, 55, 56, 57], our findings suggest that inflammation plays a key role in the FL pathogenesis. This heterogeneity in CR expression may thus reflect distinct immunobiological subtypes within FL, with potential implications for prognosis and therapeutic targeting.

In conclusion, our exploratory study identifies distinct chemokine receptor expression profiles that may drive FL progression, particularly in POD24‐cases. Increased expression of *CCR3*, *CCR4*, *CCR7*, *CXCR4*, and *XCR1* in POD24‐FLs, suggests their potential as prognostic markers for risk stratification. The clustering of most POD24 cases in a high activation‐induced CR subgroup with poor survival further supports their role in disease aggressiveness. These findings highlight the potential of the CR‐expression profile to predict outcome and response to immunochemotherapy of FL patients. However, these findings should be interpreted with caution, as they are based on a relatively small patient cohort. Larger, independent studies will be needed to validate these associations and to determine the clinical utility of CR expression profiling in predicting outcomes and guiding therapeutic decisions in FL.

## Author Contributions

Conception and design: AJAD, PN, AZ. Development of methodology: AB, JW, MSP, SH. Acquisition of data (provided animals, acquired and managed patients, provided facilities, etc.): JW; PCR; BU; KTP, FRV, MZ. Analysis and interpretation of data (e.g., statistical analysis, biostatistics, computational analysis): AZ, KP, LG, JS, JF, KTP, PN, AJAD; GS, JH, CBS. Writing, review, and/or revision of the manuscript: AZ, KP, LG, JW, AB. JS, MSP, SH, PVT, HTG. BU, JF, GS, JH, FRV, MZ, CBS, PN, KTP, AJAD. Administrative, technical, or material support (i.e., reporting or organizing data, constructing databases): SH, LG. Study supervision: AJAD, PN, HTG, KTP.

## Conflicts of Interest

The authors declare no conflicts of interest.

## Supporting information




**Table S1**: Nucleotide sequences of the forward and reverse primers used for qPCR. **Table S2**: List of antibodies that were used for immunohistochemistry. **Table S3**: Antibodies to be used for multicolour immunofluorescence staining. FITC, fluorescein isothiocyanate; DAPI, 4′,6‐diamidino‐2‐phenylindole; PE, BV, brilliant violetTM; APC, allophycocyanin; N/A, not applicable. **Table S4**: Clinicopathologic characteristics of classical follicular lymphoma (FL) patients included in this study classified based on POD24 and non‐POD24. POD24 stands for Progression of Disease within 24 months. **Table S5**: Comparison of clinicopathologic characteristics of the two clusters of our classical follicular lymphoma (FL) cohort. POD24 stands for Progression of Disease within 24 months.**Figure S1**: Poor cancer specific survival is associated with Progression of Disease within 24 months (POD24). Kaplan–Meier plots depict the cancer specific survival of our classical follicular lymphoma (FL) patient cohort (just 10 years are plotted). (a) Patients are divided in 3 groups based on the progression and/or relapse s of disease after immunochemotherapy. FL patients without any relapse after treatment (n = 18) were represented in blue; POD24‐FL cases (n = 13) are depicted in orange and relapses after the 24months (n = 21) are depicted in green. (b) Comparison between FL cases without any relapse and POD24. (c) Comparison between POD24 and relapse after 24 months, (d) Comparison between FL cases without any relapse and relapse after 24 months. e: Fl without relapse and Relapse after 24 months are considered together as non‐POD24 (n = 39). In this Kaplan‐Meier plot the non‐POD24 were compared with POD24. **Figure S2**: mRNA expression of chemokine receptor (CR) in classical follicular lymphoma (FL) and reactive tonsils as healthy control. The box plot shows mRNA expression levels of CCR2, CCR10 and CXCR2 comparing FL and reactive tonsils, patients affected by FL grades 1‐2 with grade 3a and non‐POD24 and POD24‐FLs. Values of gene expression are calculated as relative expression. POD24 stands for Progression of Disease within 24 months. **Figure S3**: mRNA expression levels of chemokine receptors (CRs) in classical follicular lymphoma (FL) based on hierarchical clustering. The dot plot shows mRNA expression levels of all 17 CRs investigated (a. for CCRs and b. for CXCRs, XCR1 and CX3CR1), patients were divided in two clusters based on the results obtained from previous hierarchical clustering analysis. Values of gene expression are calculated as relative expression. * denotes for B cell homeostastic CRs. **Figure S4**: Representative image of immunofluorescence (IF) multicolor staining of T cell subsets [CD3+, helper T cells (CD4+) and cytotoxic T cells (CD3+ CD8+)] and IHC staining of macrophages (CD68+) in follicular lymphoma patient samples. Slides were scanned using a TissueFAXS imaging system (TissueGnostics GmbH) equipped with a Zeiss Axio Imager.Z1 microscope for the IF stainings (Carl Zeiss Inc., Jena, Germany) with filters detecting DAPI, Texas Red, FITC and AF750 fluorochromes. Images were taken with Zeiss LD Plan‐Neofluar objectives (primary objective 320/0.4, ocular objective 310) at room temperature using PCO PixelFly camera (Zeiss, Oberkochen, Germany), exported from the TissueQuest software (TissueGnostics GmbH, Vienna, Austria) as tiff images, and processed in Adobe Photoshop CS5 (Adobe System, San Jose, CA). IHC staining were scanned using a Aperio AT2 scanner (200x magnification). **Figure S5**: Comparison of (a) CCR and (b) CXCR, CX3CR1, and XCR1 expression patter with immune cell content. Spearman rho correlation coefficient visualized in shades of blue to red (lower to higher).

## Data Availability

Data supporting the findings of this study are available upon reasonable request.
