# Supplementary material for: Chemokine Receptor Profiles as Predictors of Survival and Early Progression in Follicular Lymphoma
Source: EJHaem. 2025 Aug 28;6(5):e70131. doi: 10.1002/jha2.70131 (PMC12393056; doi:10.1002/jha2.70131)
Supplement: Supplementary file 1 — Table S1: Nucleotide sequences of the forward and reverse primers used for qPCR. Table S2: List of antibodies that were used for immunohistochemistry. Table S3: Antibodies to be used for multicolour immunofluorescence staining. FITC, fluorescein isothiocyanate; DAPI, 4′,6‐diamidino‐2‐phenylindole; PE, BV, brilliant violetTM; APC, allophycocyanin; N/A, not applicable. Table S4: Clinicopathologic characteristics of classical follicular lymphoma (FL) patients included in this study classified based on POD24 and non‐POD24. POD24 stands for Progression of Disease within 24 months. Table S5: Comparison of clinicopathologic characteristics of the two clusters of our classical follicular lymphoma (FL) cohort. POD24 stands for Progression of Disease within 24 months.Figure S1: Poor cancer specific survival is associated with Progression of Disease within 24 months (POD24). Kaplan–Meier plots depict the cancer specific survival of our classical follicular lymphoma (FL) patient cohort (just 10 years are plotted). (a) Patients are divided in 3 groups based on the progression and/or relapse s of disease after immunochemotherapy. FL patients without any relapse after treatment (n = 18) were represented in blue; POD24‐FL cases (n = 13) are depicted in orange and relapses after the 24months (n = 21) are depicted in green. (b) Comparison between FL cases without any relapse and POD24. (c) Comparison between POD24 and relapse after 24 months, (d) Comparison between FL cases without any relapse and relapse after 24 months. e: Fl without relapse and Relapse after 24 months are considered together as non‐POD24 (n = 39). In this Kaplan‐Meier plot the non‐POD24 were compared with POD24. Figure S2: mRNA expression of chemokine receptor (CR) in classical follicular lymphoma (FL) and reactive tonsils as healthy control. The box plot shows mRNA expression levels of CCR2, CCR10 and CXCR2 comparing FL and reactive tonsils, patients affected by FL grades 1‐2 with grade 3a and non‐POD24 and POD24 [file JHA2-6-e70131-s001.docx]

**Supplementary Material**

**Table S 1:** Nucleotide sequences of the forward and reverse primers used for qPCR.

| **Gene** | **Primer forward** | **Primer reverse** | **Probe** |
| --- | --- | --- | --- |
| ***GAPDH*** | CTTCATTGACCTCAACTACATGTTTAC | TGACAAGCTTCCCGTTCTCA | [FAM]ATGGCAAATTCCATGGCACCGTCA[TAM] |
| ***CCR1*** | CACGGACAAAGTCCCTTGGA | TGTGGTCGTGTCATAGTCCTCTGT | [FAM]TGGAGTTTCCATCCCGGCTTCTCTCT[TAM] |
| ***CCR2*** | GATGAATGGGAGTGAGGGATAGTG | GAGCCCTTTGCTTCACCTTTG | [FAM]TTTGTCCAGGCTCAGCCATGCTCA[TAM] |
| ***CCR3*** | CAACATCTACCTGCTCAACC | GCCAAAAACCCAGTTATGCC | [FAM]CCTGCTCTTCCTCGTCACCCTTCCATT TAM] |
| ***CCR4*** | TAATATTGCAAGGCAAAGACTATTCC | GCGATTTACTCCATCAGCCAGTA | [FAM]TGCAATTCCCTCTGGAGAAACCCATCA[TAM] |
| ***CCR5*** | GATTGATTTGCACAGCTCATCTG | TGTCATAGATTGGACTTGACACTTGA | [FAM]TCTCCCCGGGTGGAACAAGATGG[TAM] |
| ***CCR6*** | CAGAGCACTGCCTGAGAGTCAC | TGGTTGTAGAAAAAGGAGTGTATGGT | [FAM]AGGCAGTTCTCCAGGCTATTTGTACCGAT[TAM] |
| ***CCR7*** | GGGCACAGCCTTCCTGTG | CCACCACCAGCACGCTTT | [FAM]ACCGCCCAGAGAGCGTCATGGA[TAM] |
| ***CCR8*** | GAAGGAATTGGCAACACTGAAAC | ATCCATCAAGGCAGCGGGAC | [FAM]CCTCCAGAACAAAGGCTGTCACTAAGG[TAM] |
| ***CCR9*** | GACTTCACAAGCCCTATTCCTAACA | AAGTCAAGTGAAGTTGAAGTTAACGTAGTCT | [FAM]ACTATGGCTCTGAATCCACATCTTCCATGG[TAM] |
| ***CCR10*** | GGAGGCCACAGAAGCGGTT | GGACATCGGCCTTGTAGCAA | [FAM]AGGACGGCATACTCGGCTGAGCCACT[TAM] |
| ***CXCR1*** | CTCCTACTGTTGGACAC | ACATGTCCTCTTCAGTTTC | [FAM]CCGGTGCTTCAGTTAGATCAAACCA [TAM] |
| ***CXCR2*** | AGGTGTCCTACAGGTGAAAAG | AATCTTCAAAGCTGTCACTCTC | [FAM]CCAGCGACCCAGTCAGGATTTA[TAM] |
| ***CXCR3*** | CAGCCCAGCCATGGTCCTTG | GGAAGAGCTGAAGTTCTCCAG | [FAM[CTAAATGACGCCGAGGTTGCCGC[TAM] |
| ***CXCR4*** | CAATGACTTGTGGGTGGTTGTG | ATGCAATAGCAGGACAGGATGA | [FAM]CATGGTTGGCCTTATCCTGCCTGGTA[TAM] |
| ***CXCR5*** | CAGCCATGAACTACCCGCTAA | CCAATCTGTCCAGTTCCCAGA | [FAM]AGGTCCTCCAGGTTCTCGAGGTCCATT[TAM] |
| ***CX3CR1*** | TGACTGGCAGATCCAGAGGTT | TTCTGTCACTGATTCAGGGAACTG | [FAM]AGTCCACGCCAGGCCTTCACCA[TAM] |
| ***XCR1*** | CCATCGTGGTGGCCTACTTC | CGCAGCTCCGGATGATCT | [FAM]TCTGCAGACGCTGTTTCGGACCC[TAM] |

**Table S 2:** List of antibodies that were used for immunohistochemistry

| **Antigen** | **Host** | **Order number** | **Manufacturer** | **Dilution factor** |
| --- | --- | --- | --- | --- |
| CCR3 | Rabbit | 22351-1-AP | ProteinTech | 1:100 |
| CCR7 | Rabbit | 55425-1-AP | ProteinTech | 1:100 |
| CXCR3 | Rabbit | PA1-32503 | Invitrogen | 1:50 |
| CXCR4 | Rabbit | 11073-2-AP | ProteinTech | 1:50 |
| CXCR5 | Rabbit | 72172S | Cell Signaling | 1:50 |
| **CD68** | Mouse | M081401-2 | Agilent | ready to use |

**Table S 3:** Antibodies to be used for multicolour immunofluorescence staining*.* FITC, fluorescein isothiocyanate; DAPI, 4′,6-diamidino-2-phenylindole; PE, BV, brilliant violet^TM^; APC, allophycocyanin; N/A, not applicable.

| **Specificity** | **Conjugate** | **Clone** | **Isotype** | **Manufacturer** | **Order number** | **Dilution factor** |
| --- | --- | --- | --- | --- | --- | --- |
| CD3 | Texas Red (PE/Dazzle 594) | UCHT1 | Mouse IgG1, κ | Biolegend | 300450 | 1:100 |
| CD4 | FITC | SK3 | Mouse IgG1, κ | BD | 345768 | 1:50 |
| CD8 | Unconjugated | SK1 | Mouse IgG1, κ | BD | 346310 | 1:20 |
| Goat Anti-Mouse | Alexa Fluor 750 | Polyclonal | N/A | Invitrogen | A21037 | 1:100 |
| Nuclei | DAPI | N/A | N/A | Thermo Fisher | 10374168 | 1:10000 |

|  | **non-POD24** | **POD24** | **p-value** |
| --- | --- | --- | --- |
| **Clinicopathologic parameters** | **Patients (n= 39)** | **Patients (n=13)** |  |
|  |  |  |  |
| **Sex** | | | |
| Male | 46% (18) | 46% (6) | 0.87 |
| Female | 54% (21) | 54% (7) |  |
| **Age** | | | |
| ≤60 years | 64% (25) | 38% (5) | 0.19 |
| >60 years | 36% (14) | 62% (8) |  |
| **FLIPI** | | | |
| low | 46% (18) | 23% (3) | 0.16 |
| intermediate | 28% (11) | 23% (3) |  |
| high | 26% (10) | 54% (7) |  |
| **Grade** | | | |
| 1-2 | 54% (9) | 38% (5) | 0.23 |
| 3a | 46% (18) | 62% (8) |  |
| **Ann Arbor Stage** | | | |
| I | 26% (10) | 8% (1) | 0.79 |
| II | 5% (2) | 0% (0) |  |
| III | 28% (11) | 15% (2) |  |
| IV | 41% (16) | 77% (10) |  |

**Table S4**: Clinicopathologic characteristics of classical follicular lymphoma (FL) patients included in this study classified based on POD24 and non-POD24. POD24 stands for Progression of Disease within 24 months.

**Table S5:** Comparison of clinicopathologic characteristics of the two clusters of our classical follicular lymphoma (FL) cohort. POD24 stands for Progression of Disease within 24 months.

|  | **Cluster 1** | **Cluster 2** | **p-value** |
| --- | --- | --- | --- |
| **Clinicopathologic parameters** | **Patients (n= 19)** | **Patients (n=33 )** |  |
|  |  |  |  |
| **POD24** | | | |
| non-POD24 | 89% (17) | 67% (22) | 0.13 |
| POD24 | 11% (2) | 33% (11) |  |
| **Sex** | | | |
| Male | 47% (9) | 48% (16) | 1 |
| Female | 53% (10) | 52% (17) |  |
| **Age** | | | |
| ≤60 years | 42% (8) | 67% (22) | 0.15 |
| >60 years | 58% (11) | 33% (11) |  |
| **FLIPI** | | | |
| low | 37% (7) | 42% (14) | 0.37 |
| intermediate | 37% (7) | 18% (6) |  |
| high | 26% (5) | 39% (13) |  |
| **Grade** | | | |
| 1-2 | 47% (9) | 52% (17) | 1 |
| 3a | 53% (10) | 48% (16) |  |
| **Relapse** | | | |
| yes | 63% (12) | 67% (22) | 1 |
| no | 37% (7) | 33% (11) |  |
| **Ann Arbor Stage** | | | |
| I | 21% (4) | 21% (7) | 0.79 |
| II | 5.3% (1) | 3% (1) |  |
| III | 32% (6) | 21% (7) |  |
| IV | 42% (8) | 55% (18) |  |
| **Survival** | | | |
| alive | 84% (16) | 58% (19) | 0.096 |
| dead | 16% (3) | 42% (14) |  |


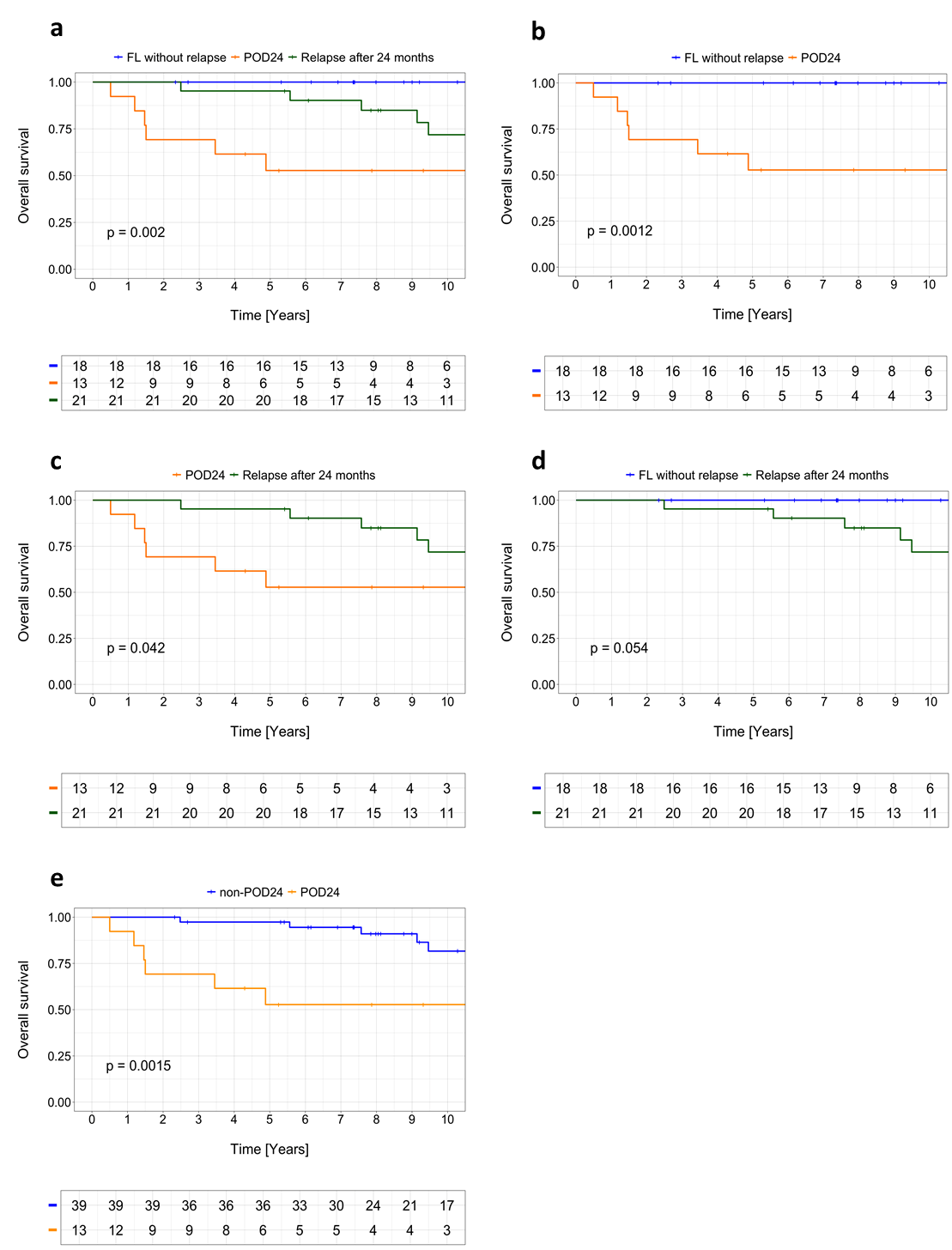


**Supplementary Figure S1:** Poor cancer specific survival is associated with Progression of Disease within 24 months (POD24). Kaplan–Meier plots depict the cancer specific survival of our classical follicular lymphoma (FL) patient cohort (just 10 years are plotted). (a) Patients are divided in 3 groups based on the progression and/or relapse s of disease after immunochemotherapy. FL patients without any relapse after treatment (n=18) were represented in blue; POD24-FL cases (n=13) are depicted in orange and relapses after the 24months (n=21) are depicted in green. (b) Comparison between FL cases without any relapse and POD24. (c) Comparison between POD24 and relapse after 24 months, (d) Comparison between FL cases without any relapse and relapse after 24 months. e: Fl without relapse and Relapse after 24 months are considered together as non-POD24 (n=39). In this Kaplan-Meier plot the non-POD24 were compared with POD24.


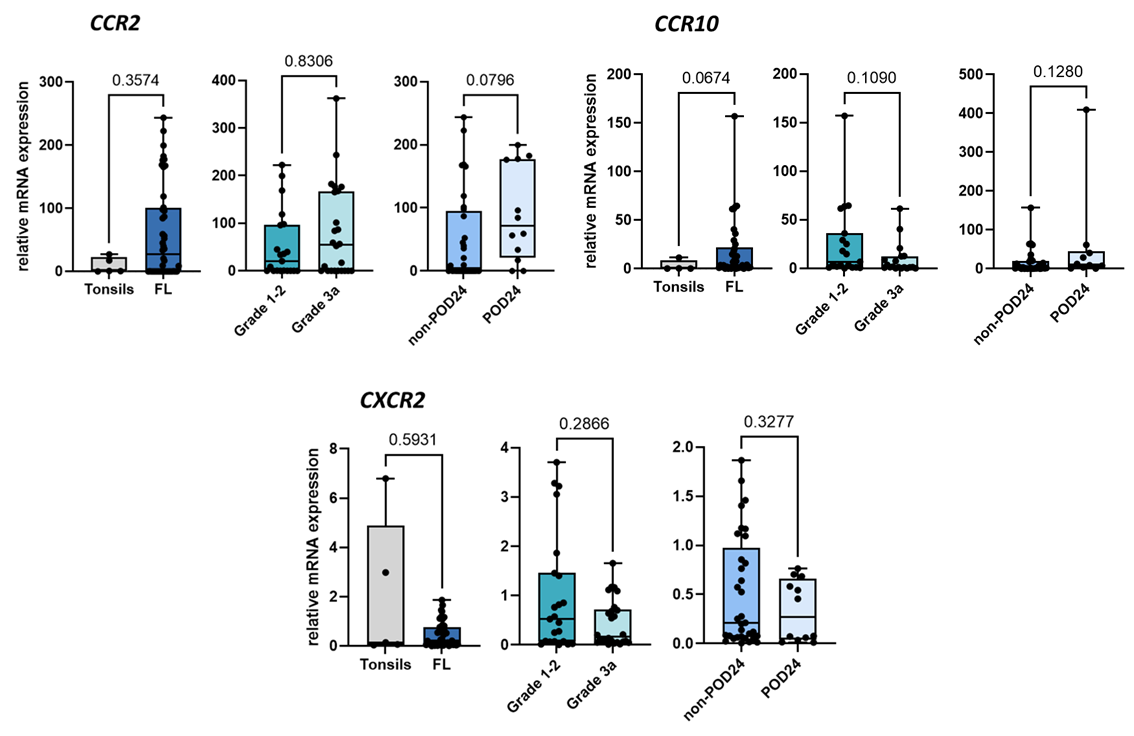


**Supplementary Figure S2:** mRNA expression of chemokine receptor (CR) in classical follicular lymphoma (FL) and reactive tonsils as healthy control. The box plot shows mRNA expression levels of *CCR2*, *CCR10* and *CXCR2* comparing FL and reactive tonsils, patients affected by FL grades 1-2 with grade 3a and non-POD24 and POD24-FLs. Values of gene expression are calculated as relative expression.  POD24 stands for Progression of Disease within 24 months.


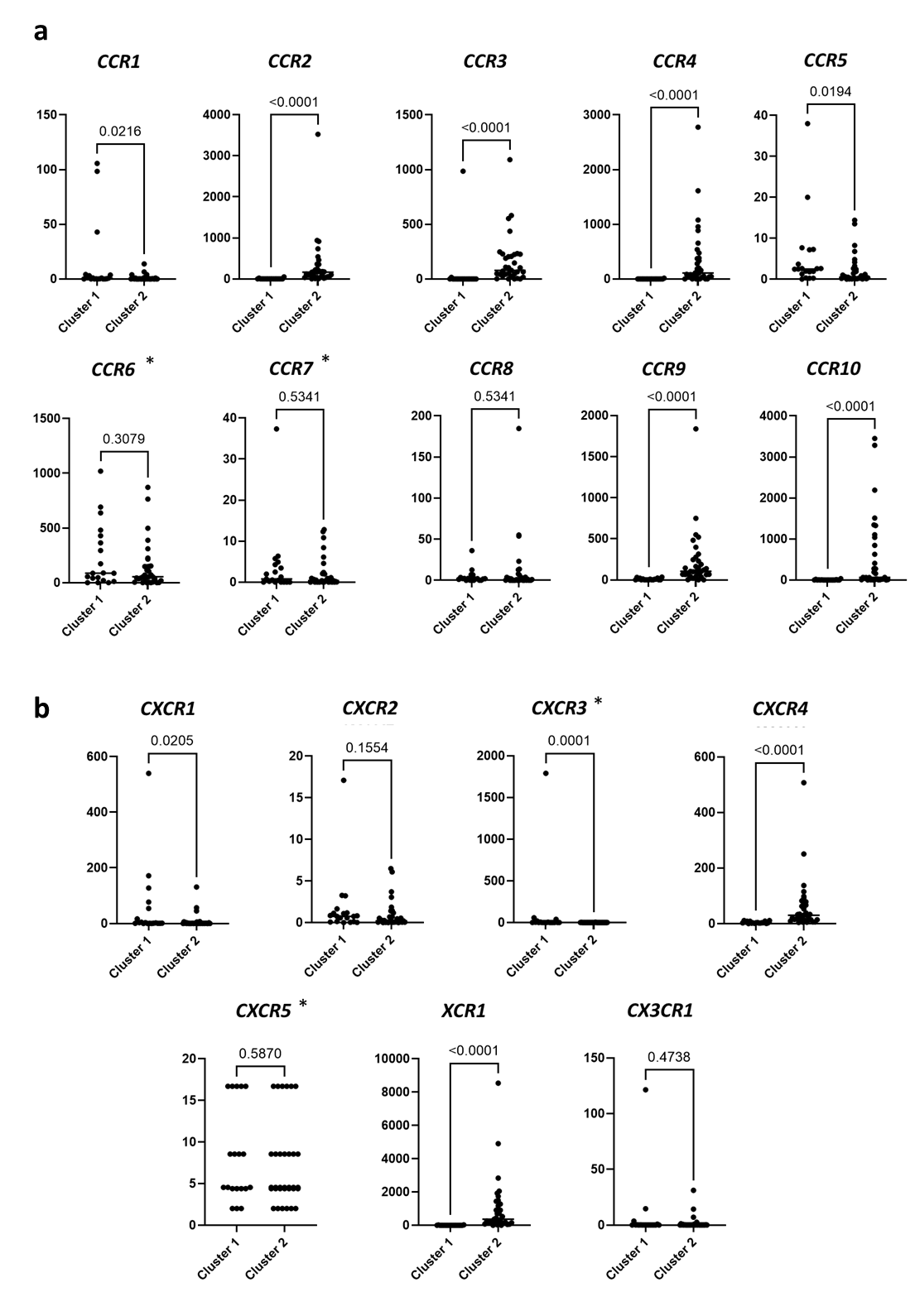


**Supplementary Figure S3**: mRNA expression levels of chemokine receptors (CRs) in classical follicular lymphoma (FL) based on hierarchical clustering. The dot plot shows mRNA expression levels of all 17 CRs investigated (a. for CCRs and b. for CXCRs, XCR1 and CX3CR1), patients were divided in two clusters based on the results obtained from previous hierarchical clustering analysis. Values of gene expression are calculated as relative expression. * denotes for B cell homeostastic CRs.

**Supplementary Figure S4:** Representative image of immunofluorescence (IF) multicolor staining of T cell subsets [CD3+, helper T cells (CD4+) and cytotoxic T cells (CD3+ CD8+)] and IHC staining of macrophages (CD68+) in follicular lymphoma patient samples. Slides were scanned using a TissueFAXS imaging system (TissueGnostics GmbH) equipped with a Zeiss Axio Imager.Z1 microscope for the IF stainings (Carl Zeiss Inc., Jena, Germany) with filters detecting DAPI, Texas Red, FITC and AF750 fluorochromes. Images were taken with Zeiss LD Plan-Neofluar objectives (primary objective 320/0.4, ocular objective 310) at room temperature using PCO PixelFly camera (Zeiss, Oberkochen, Germany), exported from the TissueQuest software (TissueGnostics GmbH, Vienna, Austria) as tiff images, and processed in Adobe Photoshop CS5 (Adobe System, San Jose, CA). IHC staining were scanned using a Aperio AT2 scanner (200x magnification).


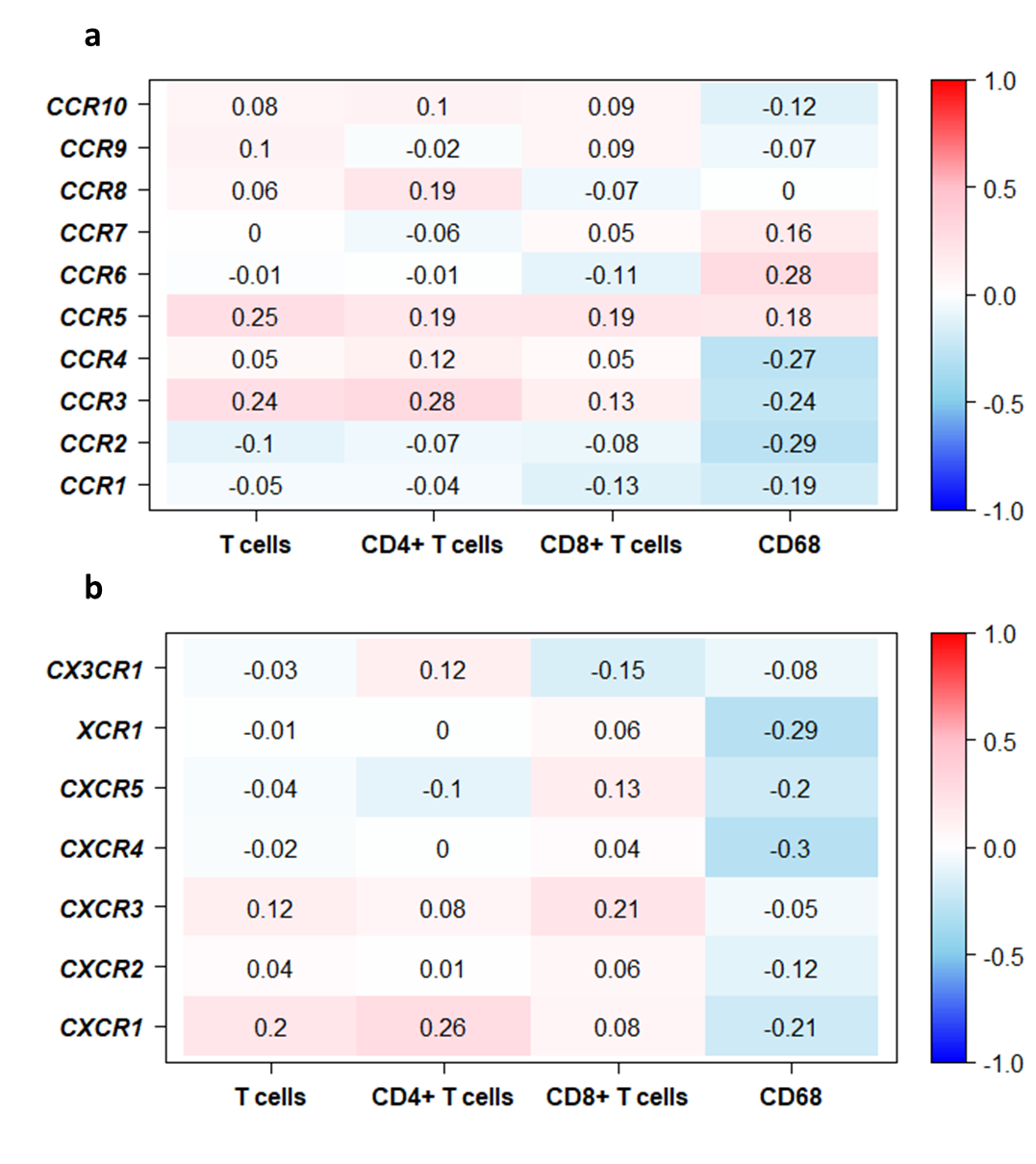


**Supplementary Figure S5:** Comparison of (a) *CCR* and (b) *CXCR, CX3CR1,* and *XCR1* expression patter with immune cell content. Spearman rho correlation coefficient visualized in shades of blue to red (lower to higher).
